# Tuberculosis and Lung Cancer: Insights from a Narrative Review

**DOI:** 10.3390/cancers18010083

**Published:** 2025-12-27

**Authors:** Antonio-Andrei Cotea, Ancuta-Alina Constantin, Florin-Dumitru Mihaltan

**Affiliations:** 1 Department of Cardio-Thoracic Pathology, “Carol Davila” University of Medicine and Pharmacy, 050474 Bucharest, Romania; 2 National Institute of Pneumophthisiology “Marius Nasta”, 050159 Bucharest, Romania

**Keywords:** lung cancer, tuberculosis, chronic inflammation, immune checkpoint inhibitors, diagnostic overlap

## Abstract

The interaction between LC and active TB has been known for many years. The first description of ‘cancerous phthisis’ was reported by Bayle in 1810. The results of subsequent attempts to establish an etiological correlation between the two clinical entities have ranged from insignificant—even antagonistic—to a direct causal relationship. This narrative review explores the relationship between tuberculosis and LC assessing overall and site-specific malignancy risks in individuals with TB compared with the general population. We also examine the timing of cancer development following TB, highlighting periods of elevated risk. Chronic inflammation, immune dysregulation, and metabolic changes associated with TB may contribute to tumor initiation and progression, with organ-specific factors influencing susceptibility to cancers such as lung, head and neck, gastrointestinal, and hematologic malignancies. Clinically, awareness of these interactions supports targeted TB screening in high-risk cancer patients, careful monitoring for TB reactivation during cancer therapy, and consideration of immunological and metabolic factors when planning treatment.

## Simple Summary

Tuberculosis (TB) can cause long-lasting changes in the lungs, and these changes may increase the chance of developing lung cancer (LC). Although this possible link has been discussed for many years, it remains unclear how strong the relationship truly is or why it occurs. This review brings together current knowledge on how TB may influence LC risk, including when LC is most likely to appear after TB and what biological processes may connect the two conditions. We describe how ongoing inflammation, altered immune responses, and structural damage in the lungs caused by TB could help cancer develop. By clarifying these connections, this work aims to support researchers and clinicians in better identifying people at higher risk and improving strategies for prevention, early diagnosis, and patient care.

## 1. Introduction

TB is an ancient disease that has impacted humans for over 8000 years and remains one of the top ten causes of death worldwide. Today, with prompt diagnosis and appropriate treatment, most cases of TB are fully curable [1]. However, TB continues a major global health concern, with many cases undiagnosed or untreated. Current strategies for managing latent and active TB may be insufficient, prompting interest in host-directed therapies that boost immune responses and reduce inflammation [2].

Over 10 million people develop TB each year, and by 2020 there were an estimated 155 million survivors worldwide. These individuals face higher all-cause mortality and shorter life expectancy, partly due to increased rates of non-communicable diseases (NCDs), including cancer—the second leading cause of death after cardiovascular disease in this group [3].

Infectious pathogens are known carcinogens, contributing to an estimated 2.2 million cancers annually worldwide [3]. Metabolic pathways involved in TB pathogenesis may also contribute to LC progression, offering potential targets for host-directed therapies [2]. TB infection has long been suspected to increase cancer risk, particularly among individuals with additional risk factors such as smoking, immune suppression, or alcohol misuse. While recent reviews link TB to both pulmonary and extrapulmonary cancers, screening guidelines for TB survivors remain limited. Understanding cancer incidence and timing after TB could inform better clinical practice and screening strategies [3].

Infections cause 10% of new cancer cases globally, while cancer itself raises infection risk. With advances in treatment, broader use of immunotherapy, and longer patient survival, TB and cancer are increasingly seen together in clinical practice [4].

The co-occurrence of TB and lung cancer is well-documented. Three explanations have been proposed: TB increases LC risk; LC reactivates latent TB; or both occur together by chance. LC can weaken immunity and invade old TB lesions, leading to reactivation. Although coexistence may be coincidental in high-prevalence regions, emerging evidence suggests a potential causal relationship [5].

Researchers are studying strategies to decrease lung cancer risk by either reducing ROS levels or improving the treatment of Mycobacterium tuberculosis infections, including the use of antioxidants and the development of more effective anti-TB drugs or vaccines [4].

This narrative review aims to summarize the bidirectional relationship between tuberculosis and lung cancer, highlighting epidemiology, risk factors, clinical consequences, and practical management strategies in the era of modern therapies.

### Methods

We conducted a narrative review of studies investigating TB in lung cancer patients. Literature searches were performed in PubMed, Scopus, and Web of Science for articles published from 2000 to 2025, using combinations of the keywords “tuberculosis,” “latent TB,” “lung cancer,” “risk factors,” and “epidemiology.” Only English-language studies reporting TB incidence, risk factors, or clinical outcomes in LC patients were included. Data were extracted on study design, population characteristics, TB type, cancer type, follow-up period, and key findings. Results were synthesized narratively, with emphasis on regional differences and clinical implications.

## 2. Bidirectional Epidemiology: TB and Cancer Risk

In 2019, there were an estimated 2.3 million new tracheal, bronchus, and lung (TBL) cancer cases and 2 million deaths worldwide. From 1990 to 2019, incidence fell by 2.6% and mortality by 7.8%, while disability-adjusted life years (DALYs) declined by 16.2%. Greenland, Monaco, and Montenegro showed the highest rates, whereas Honduras and Cabo Verde recorded the greatest increases. Rates were consistently higher in men and rose with age. Globally, smoking (62%), air pollution (15%), and high fasting plasma glucose (10%) were the leading risk factors for TBL cancer DALYs [6].

TB remains the leading infectious killer and was the ninth leading cause of death in 2016. Despite declining mortality, 10.4 million new TB cases occurred that year. According to the World Health Organization (WHO), the Sustainable Development Goals include the complete elimination of the tuberculosis epidemic by 2030. Evidence links TB to increased cancer risk, yet its global cancer burden remains unquantified. Estimating cancer cases attributable to TB could inform health priorities, especially in countries facing both diseases [7].

Using Taiwan’s national insurance data (1998–2000), Yu et al. identified 4480 adults with newly diagnosed tuberculosis from a cohort of over 700,000 cancer-free individuals aged over 20. They tracked LC incidence through 2007, finding it to be 11 times higher in the TB group (Table 1), with an adjusted hazard ratio of 3.32 (95% CI 2.70–4.09) after accounting for factors such as chronic obstructive pulmonary disease (COPD) and smoking-related cancers [8]. In China, where TB is common, the population attributable fraction (PAF) was 12.67%, compared with 0.62% for COPD. Even in North America, where tuberculosis prevalence was lowest, the PAF was 1.14%, exceeding those of COPD (0.40%) and pneumonia (0.17%) [9].

Incidence and Risk of LC in Patients with TB

Cigarette smoking has been a major cause of lung cancer since 1912. Other contributing factors include air pollution, diet, occupational exposure, and a family history of cancer. Recent advances in molecular diagnostics have also enabled studies on genetic and inflammatory factors involved in lung carcinogenesis [10]. Furthermore, evidence suggests that in diabetes, the lung may be affected by microangiopathy, increasing vulnerability to infections and pulmonary dysfunction. Moreover, diabetes-related factors and established lung cancer risks such as smoking and chronic lung disease may jointly promote oxidative stress, chronic inflammation, and accelerated pulmonary aging, thereby increasing lung cancer risk [11].

Growing evidence (Table 1) suggests that TB may increase the risk of lung cancer, extending its health burden beyond the period of active disease. A meta-analysis of 73 studies found that individuals with prior TB had a significantly higher likelihood of developing LC, with adjusted hazard and odds ratios ranging from 1.5 to 1.7 (Table 1). The risk was greatest within two years of TB diagnosis, indicating a period of heightened vulnerability or possible diagnostic overlap. Although causality remains uncertain due to confounding and study design limitations, these findings underscore the need for vigilant follow-up of TB survivors and prospective research to identify high-risk groups [12].

Studies assessing LC risk in TB patients typically compared TB cohorts with general or non-TB populations, sometimes including COPD subgroups. Reported incidence rate ratios (IRRs) varied with study duration: the highest IRR—11—fold greater risk—was observed in a Taiwanese cohort over nine years (26.3 vs. 2.41 per 10,000), while the lowest was 1.76 over ten years (Table 1). Overall, shorter follow-up periods tended to yield higher IRRs [13].

A systematic review by Cabrera-Sánchez et al. similarly reported a modest but consistent increase in LC risk following TB, with hazard and odds ratios between 1.5 and 2 across analyses (Table 1). While definitive causality cannot be confirmed, the association is biologically plausible. Chronic lung inflammation—driven by macrophage-derived cytokines and reactive nitrogen species—may induce DNA damage, genetic mutations, and angiogenesis, fostering carcinogenesis. This mechanism is supported by findings in mycobacterium-infected animal models [12].

COPD is a well-established risk factor for LC, and smoking remains the leading cause of both conditions through sustained systemic inflammation. TB further elevates LC risk, particularly in regions with moderate to high TB prevalence where TB frequently coexists with COPD. Chronic inflammation likely represents the common pathogenic link among these diseases [14].

**Table 1:** Shows evidence that prior tuberculosis increases the risk of developing lung cancer, summarizing study designs, key findings, and available numerical risk estimates.

Ref.	Study	Design/Population	Key Findings (TB → LC)	Notes
[8]	Vento & Lanzafame, 2011	Review	TB-driven chronic inflammation can promote carcinogenesis.	Mechanistic review; no numerical data.
[8]	Sisti & Boffetta, 2012	Risk factors in never-smokers	Prior TB listed as contributor to LC in never-smokers.	TB considered among minor but relevant risk factors.
[12]	Cabrera-Sánchez et al., 2022	Systematic review + meta-analysis	LC risk is higher after TB; pooled HR/OR ~1.5–1.7.	Most comprehensive quantitative synthesis.
[13]	Bhowmik et al., 2022	Literature review	Consistently higher LC incidence in TB patients across studies.	Summarizes last decade’s TB—LC data.
[14]	Park et al., 2022	COPD cohort with/without TB	Prior TB raises LC incidence compared to COPD-only controls.	TB identified as independent LC risk factor.
[6]	Safiri et al., 2021	Global burden analysis	TB included among global LC-attributable risk factors.	Provides population-attributable estimates.

Incidence and Risk of TB in Patients with LC

All studies investigating the risk of active or latent TB in lung cancer patients (Table 2) have recruited lung cancer individuals as the case groups, with general or non-lung cancer populations (which may include other diseases) serving as controls [13]. Mata-Marín et al. reported a high prevalence of latent tuberculosis infection (~34%) among patients with immune-mediated inflammatory diseases treated with disease-modifying antirheumatic drugs. Prior TB exposure was the strongest risk factor for LTBI, highlighting the need for routine TB screening in immunosuppressed patients to prevent reactivation [15].

A Canadian cohort reported that migrants with cancer had an increased risk of active TB after adjustment for age, sex, TB incidence in the country of origin, immigration classification, contact status, and comorbidities. The highest risk was observed in lung cancer and sarcoma, followed by leukemia, lymphoma and gastrointestinal cancers. Most active TB cases (65.9%) were diagnosed more than six months after cancer diagnosis [16].

Furthermore, a Thai study using the Khon Kaen population-based cancer registry and TB databases investigated TB risk among 40,948 cancer patients from 2001 to 2015. During follow-up, 472 patients developed TB after cancer diagnosis (cumulative incidence 1.15%). In a sub-cohort of nine cancer types (*n* = 9733), 206 TB cases were identified (cumulative incidence 2.11%). Risk factors for TB included male sex, age over 70, and primary cancer site, with lung cancer showing the highest risk compared to thyroid cancer. Cancer stage and treatment were not significantly associated with TB risk. The study concluded that older age, male sex, and certain cancer types are independent risk factors, highlighting the potential value of targeted latent TB screening in high-risk groups [17].

Two Taiwanese studies examined TB risk in lung cancer patients. In a cohort of 5406 squamous cell carcinoma patients, TB and other pulmonary comorbidities modestly increased all-cause mortality, with the highest risk in men with asthma, COPD and TB [18]. Stage III–IV male patients with coexisting TB also showed elevated mortality, while early-stage female patients with asthma had lower risk. In a separate study of 340 newly diagnosed lung cancer patients, 28.2% had latent TB infection (LTBI). COPD, fibrocalcified lesions, and tumors in typical TB areas were independent predictors of LTBI. Indeterminate QFT-GIT results, low BMI, and advanced cancer stage were associated with higher 1-year mortality, while survival was similar between LTBI and non-LTBI patients [19].

Risk factors for chronic pulmonary infection (CPI) include fibronodular lesions, bronchiectasis, COPD, and sequelae of pulmonary TB. Although a causal relationship between CPI and lung cancer has not been definitively established, patients with CPI frequently have a history of LC. A meta-analysis reported that LC was associated with a sixfold higher risk of developing post-TB pulmonary sequelae (Table 2), making it the solid tumor most frequently associated with TB-related pulmonary disease [20].

Immune checkpoint inhibitors (ICIs) have greatly improved LC outcomes but are associated with a significant risk of serious infections (Table 2). In a retrospective study of 710 LC patients treated with ICIs, 26.9% developed severe infections—most commonly pulmonary. While bacterial infections predominated, Mycobacterium tuberculosis accounted for 6.5% of cases, emphasizing the strong clinical connection between LC and TB. Identified risk factors included COPD, asthma, systemic glucocorticoid use, lymphopenia, and altered CD4/CD8 ratios. These findings highlight the importance of comprehensive infection risk assessment and routine TB screening before and during ICI therapy [21].

**Table 2:** Shows evidence that patients with lung cancer have an increased risk of developing or reactivating tuberculosis, including factors such as immune suppression, radiotherapy, and cancer therapies.

Ref.	Study	Design/Population	Key Findings (LC → TB)	Notes
[8]	Vento & Lanzafame, 2011	Review	LC increases TB reactivation risk due to immunosuppression.	Emphasizes bidirectional nature of relationship.
[13]	Bhowmik et al., 2022	Literature review	LC patients lowers immunity, which results in higher TB incidence.	Summarizes chemotherapy, RT, malnutrition effects.
[20]	Choi et al., 2023	LC post-radiotherapy cohort	Chronic infections after RT; TB appears among reported infections.	TB not dominant but clinically relevant.
[21]	Wang et al., 2025	LC on immune checkpoint inhibitors	Serious infections raises under ICI therapy; TB a recognized risk.	ICI-related immune shifts may enable TB.
[7]	Sharma & Khubchandani, 2024	Global LC burden analysis	Infectious comorbidities, including TB, cluster in high-TB regions.	Contextual global epidemiology.

## 3. Pathophysiological and Immunological Links

Both *Mycobacterium tuberculosis* (Mtb) and cancer employ complex, coordinated strategies to evade the immune system, disrupting both innate and adaptive immune responses. A key feature of both tuberculosis granulomas and the tumor microenvironment (TME) is the altered macrophage polarization and T cell exhaustion that drive disease persistence. In both conditions, early recognition by macrophages is critical for initiating the immune response [22].

Following Mtb infection, the immune response is initiated by key cytokines such as tumor necrosis factor-alpha (TNF-α) and type II interferon (IFN-γ). TNF plays a central role in activating macrophages—the primary cells responsible for engulfing and attempting to eliminate Mtb. Activated macrophages produce reactive nitrogen and oxygen intermediates that inhibit or kill the bacteria. TNF is also crucial for the formation and maintenance of granulomas, organized immune structures that contain the infection but can also serve as reservoirs for dormant bacteria [23]. As the granuloma matures, a fibrous cuff develops around the macrophage layer, and lymphocytes form follicle-like structures encircling it, isolating infected macrophages and the necrotic core containing the bacteria. This stage marks latent infection, during which bacterial growth is contained [22]. The interplay of TNF and IFN-γ is essential for controlling TB, with insufficient activity allowing bacterial proliferation and excessive activity driving tissue-damaging immunopathology [23].

Macrophages and Trained Immunity

TB vaccine research centers on Bacille Calmette-Guérin (BCG), a live attenuated Mycobacterium bovis strain and early cancer immunotherapy. BCG activates innate immunity, particularly macrophages, contributing to its preventive and therapeutic effects. However, it poorly prevents pulmonary TB in humans, with protection against disseminated TB waning by adolescence. Efficacy varies geographically, likely due to environmental mycobacteria. In rodents, BCG protects against TB and other diseases, and subcutaneous vaccination provides rapid, T cell–independent protection [22].

Alveolar macrophages are frontline immune cells in *Mycobacterium tuberculosis* infection, involved in pathogen defense, antigen presentation, and immune regulation. They have dual roles: some subsets actively restrict Mtb, while others can serve as permissive niches that support bacterial survival, reflecting their functional heterogeneity. Mtb uses unique surface lipids and effectors to evade innate killing and persist within alveolar macrophages. Alveolar macrophages also interact with other immune cells to help balance host immunity, and disruption of this balance can worsen tuberculosis [24].

Tissue-resident macrophages in the lung are key defenders against pathogens and link innate and adaptive immunity, also influencing carcinogenesis and cancer immunotherapy. In lung tumors, infiltrating macrophages—called tumor-associated macrophages (TAMs)—are the dominant immune cells in the tumor microenvironment. TAMs develop along different pathways: classical activation gives rise to pro-inflammatory M1 macrophages, while alternative activation produces anti-inflammatory, tumor-supporting M2 macrophages. These TAM subsets are strongly involved in tumor progression, promoting invasion, metastasis, immunosuppression, and resistance to therapy, and are emerging as promising targets for immunotherapy [25].

In the lung cancer microenvironment, many macrophages acquire immunosuppressive traits and express high levels of PD-L1, which engages PD-1 on T cells and weakens CD8^+^ cytotoxic responses, thereby inhibiting effective anti-Mycobacterium tuberculosis immunity and suppressing adaptive immune defense against TB. Moreover, *M. tuberculosis* can evade macrophage killing by blocking phagosome-lysosome fusion, allowing the bacteria to survive and replicate within macrophages—a mechanism that contributes to the development of active tuberculosis in patients with lung cancer [26].

Circular RNAs in TB and Lung Cancer

Circular RNAs (circRNAs) are a class of stable, non-coding RNAs with covalently closed-loop structures that resist exonuclease degradation. They regulate gene expression and are increasingly recognized for their roles in lung cancer and tuberculosis. By acting as “molecular sponges,” circRNAs sequester microRNAs (miRNAs), modulating downstream pathways and promoting cancer progression. Unlike linear RNAs, they are non-immunogenic, require no nucleoside modifications, and their strong miRNA-binding capacity renders them promising diagnostic and therapeutic targets [27].

CircRNAs are important regulators of inflammatory responses during *Mycobacterium tuberculosis* infection. They can modulate host immunity by controlling the expression of inflammation-related genes and signaling pathways. For example, circ-ZNF277 sponges miR-378d to activate NF-κB signaling, increasing pro-inflammatory cytokines such as IL-1β, IL-6, and TNF-α and helping restrict *M. tuberculosis* survival in macrophages. CircRNAs can also influence the strength and duration of inflammation by interacting with microRNAs and other regulatory molecules to adjust cytokine release and immune signaling, highlighting their role in host defense and their potential as therapeutic targets in tuberculosis [28].

Lung cancer (LC), the most prevalent malignancy worldwide and the leading cause of cancer-related mortality, often results in death through metastasis via the bloodstream or lymphatic system. Tumor-derived exosomes (TDEs) play a central role in this process by reshaping the tumor microenvironment and establishing pre-metastatic niches. Exosomal circRNAs (exo-circRNAs), a subset of circRNAs enclosed within exosomes, can be transferred to distant cells where they modulate the tumor microenvironment (TME), facilitating cancer cell proliferation, invasion, and metastasis [29].

CircRNAs actively remodel the tumor microenvironment—a critical driver of cancer progression—by influencing processes such as epithelial-stromal transformation, tumor angiogenesis, immune cell activity, and inflammatory signaling. Immune cells are among the most abundant tumor microenvironment components and play a major role in controlling cancer cell growth [30].

Aberrant circRNA expression has also been associated with infectious diseases such as tuberculosis. Several studies have identified distinct circRNAs, including hsa_circ_103571, uniquely expressed in active TB cases—previously unreported in human disease—highlighting their diagnostic potential. Although the use of endogenous molecules like circRNAs in infectious disease detection is still in its early stages, their stability, specificity, and functional relevance make them promising biomarkers for TB diagnosis [31].

Role of latent TB infection in cancer susceptibility.

Tuberculosis is a chronic infectious and inflammatory disease, and its link to subsequent cancer development has been explored for decades. Systematic reviews have demonstrated an association between tuberculosis and increased cancer risk, while recent population-based studies suggest that tuberculosis may elevate the risk of lung cancer as well as other malignancies such as esophageal and head and neck cancers. The strong relationship observed between tuberculosis and lung cancer is thought to be mediated, at least in part, by chronic inflammation and infection associated with tuberculosis, which may contribute to carcinogenic processes [32].

A systematic review and meta-analysis of 49 studies including 52,480 cancer patients evaluated the relationship between tuberculosis and cancer incidence across 17 cancer sites. The analysis demonstrated that tuberculosis was associated with an increased risk of cancer at ten sites in adults. It was further estimated that, in 2015, tuberculosis accounted for approximately 2.93% (1.45–4.75%) of all cancers in men and 1.61% (0.78–2.67%) in women across 195 countries and territories [33].

Cancer has long been recognized as a risk factor for the development of active Mycobacterium tuberculosis infection, a relationship noted since the 1970s. However, the absolute and relative risks associated with specific cancer types, as well as how these risks have evolved over time, remain insufficiently defined [34]. In a study of patients with newly diagnosed primary bronchogenic carcinoma, latent tuberculosis infection (LTBI) was identified in 25% of cases, while 12.5% had indeterminate QuantiFERON-TB results. Smoking was significantly associated with a higher prevalence of LTBI, whereas comorbidities, tumor site, and histopathology showed no correlation. These findings highlight a notable coexistence of LTBI in LC patients and underscore the need for further research on the impact of LTBI treatment on clinical outcomes and prognosis [35]. A case–control study by Mortezazadeh et al. found no significant difference in latent tuberculosis prevalence between cancer patients (27.1%) and controls (20.7%). However, cancer patients with larger tuberculin skin test (TST) reactions and latent infection showed higher mortality, suggesting that LTBI may adversely affect prognosis despite no direct link to cancer incidence [36].

## 4. Clinical and Diagnostic Overlap

Lung involvement in TB is highly heterogeneous, both in the degree of functional impairment and the types of ventilatory defects observed. Pulmonary function can range from normal to severely reduced, reflecting the variable impact of the disease. Patients may present with cavitation, fibrosis, or nodular infiltrates, and often a combination of these pathologies (Table 3), highlighting the complex and multifaceted nature of TB-related lung damage [37].

Lung cancer frequently presents as pulmonary nodules on CT, which are broadly classified by density into solid and subsolid nodules with differing morphology and pathology. Solid nodules tend to have a higher malignant potential and worse prognosis than subsolid ones, and accurate early identification is crucial. Key CT features linked to malignancy include spiculation, lobulation, vascular convergence, and pleural retraction, which help distinguish malignant from benign nodules, though these signs are often absent in smaller lesions, complicating diagnosis (Table 3). Understanding how these imaging features vary with nodule size can improve early detection of potentially malignant solid nodules [38].

Conventional CT scanning is the primary method for distinguishing TB from LC (Table 3), based on lesion location, size, shape, lobe involvement, borders, density, and enhancement patterns. Traditional qualitative assessment relies on these imaging features but is subjective, experience-dependent, and lacks quantitative measures, making reproducibility limited. Therefore, definitive diagnosis often requires biopsy and histopathological confirmation [39].

TB is recognized as a diagnostic chameleon that can mimic malignancy. In the thorax, it may present as pulmonary infiltrates and/or mediastinal lymphadenopathy. In countries with low TB incidence but high LC rates, its variable clinical presentation often leads to misdiagnosis, resulting in delayed treatment and unnecessary diagnostic procedures [40].

TB and LC can be difficult to distinguish, as both may show high metabolic activity on 18-FDG-PET (Table 3) and similar radiological features [40]. Other non-malignant conditions that can also cause increased 18F-FDG uptake include tuberculoma, acute and chronic pneumonia, abscesses, fungal infections, sarcoidosis, parasitic infestations, and pneumoconiosis, emphasizing the need for careful differential diagnosis [41].

Differentiating TB from LC remains a significant diagnostic challenge (Table 3). Historical studies have documented instances in which TB was identified among patients initially suspected of having cancer: Prytz et al. reported 91 such cases; Pitlik et al. identified 26 cases among 70,000 referrals; and Rolston et al. found 37 infections (27% of which were mycobacterial) among 2908 patients with suspected LC. These findings highlight the limitations of imaging-based diagnosis and emphasize the necessity of microbiological or pathological confirmation to prevent unnecessary interventions and associated complications [40].

Asymptomatic pulmonary tuberculosis (PTB) can mimic LC on imaging, presenting abnormal CT findings and increased glucose uptake. Risk factors include age < 60, male sex, diabetes, spiculated margins, and lower SUVmax, with an optimal cut-off of 8.45 for differentiation. Accounting for these factors alongside imaging may improve diagnostic accuracy and PET-CT specificity, particularly in TB-endemic regions [42].

## 5. Therapeutic Interactions

Treatment of co-existent TB and lung cancer

Diagnosing concurrent TB and LC remains challenging, as symptoms and imaging findings often overlap, and granulomatous inflammation seen in resected lung tissue is not specific to TB [5]. Primary treatments for lung cancer include surgery, chemotherapy, radiotherapy, and immunotherapy (if IGRA is negative), while tuberculosis requires a long-term course of anti-TB therapy [43]. Moreover, standard LC treatments—including chemotherapy and immunotherapy—can influence both the risk and management of TB [5].

In a retrospective study by Tamura et al., 39 patients developed TB during LC treatment. Most were elderly male smokers with additional risk factors, including prior TB or corticosteroid use. Notably, latent TB infection was diagnosed in only one patient, and none received LTBI therapy. TB emerged across diverse clinical contexts, including during active treatment, post-treatment follow-up, and best supportive care, highlighting the complex interplay between LC, its management, and TB reactivation in high-risk populations [44].

Immune checkpoint inhibitors (ICIs) are first-line therapy for advanced NSCLC, but their safety may be altered in patients with active or latent TB. TB infection can occur during ICI therapy, and simultaneous anti-TB and ICI treatment has been reported with favorable outcomes. Practical management includes screening for latent TB before immunotherapy, considering prophylactic treatment in high-risk patients, and monitoring for drug—drug interactions between anti-TB and anticancer agents [45].

In early-stage LC, patients are first screened for TB using PCR and CT scans. If TB is present, anti-TB therapy is initiated, with surgery reserved for those who do not improve; otherwise, surgery is performed before starting anti-TB medication. For metastatic LC, chemotherapy or immunotherapy is used based on the patient’s status, though immunotherapy is typically avoided in active TB cases to prevent reactivation. While PD-1/PD-L1 inhibitors are effective in patients with latent TB, they carry risks of TB-related mortality and reactivation. Surgical options like pneumonectomy, lobectomy, or segmentectomy remain vital for treating coexistent LC and TB, often alongside concurrent or sequential medical therapies [43].

Impact of cancer therapy on TB reactivation risk.

LC patients have a high risk of TB reactivation, driven by both the disease and its treatments. Cytotoxic chemotherapy, targeted therapies, and especially PD-1/PD-L1 inhibitors increase this risk, with immune checkpoint blockade linked to up to 35-fold higher TB incidence. LC and its therapies together amplify TB activation, though further studies are needed to clarify mechanisms [46].

One review by Saeed ASH et al. highlights that solid cancer patients, especially those receiving chemotherapy, are at increased risk of latent TB reactivation. Lung and head-and-neck cancers show particularly high risk, and concurrent anticancer and anti-TB therapy can be safe [47].

Immune checkpoints are vital for maintaining peripheral tolerance and preventing excessive immune responses that lead to immunopathology. While these molecules can hinder effective anti-tumor immunity, their inhibition via monoclonal antibodies—such as pembrolizumab (anti-PD-1) and ipilimumab (anti-CTLA-4)—has become a powerful clinical tool. By blocking these pathways, clinicians can re-invigorate anti-tumor T-cells, enabling the effective targeting and eradication of malignant cells [48].

Although anticancer immunotherapy enhances immunity, it can paradoxically increase tuberculosis risk. Anti-PD-1 therapy may disrupt T-cell balance, dysregulate cytokines such as TNF-α, and destabilize granulomas, facilitating *M. tuberculosis* proliferation. Rapid T-cell activation can also promote extracellular matrix destruction and recruit inflammatory cells, suggesting that excessive immunity may be as detrimental as immune deficiency, akin to IRIS in HIV patients on antiviral therapy [49]. Symptoms like weight loss and fever may be misattributed to cancer progression, so screening for latent or active TB is advised before starting ICIs, especially in high-risk patients. Management generally follows TNF-α inhibition protocols: temporarily hold ICIs, stop other immunosuppression, and start anti-TB therapy. Timing to safely resume ICIs is unclear, with 2–4 weeks suggested. Further research is needed to optimize TB prevention during ICI therapy [50]. While T-cells are essential for tuberculosis immunity, the progression of the disease despite enhanced T-cell activity following immune checkpoint inhibition remains a significant clinical paradox. This negative impact on TB immunity highlights a critical immunological parallel: the same pathways that are successfully manipulated to treat cancer also fundamentally regulate the host response to *M. tuberculosis*. Furthermore, because immune checkpoints are expressed across various cell types, the clinical consequence of TB reactivation may stem from broader systemic effects beyond simple T-cell modulation [48].

For the past decade, immune checkpoint inhibitors have been a standard immunotherapy for malignancies like lung cancer. However, reports link this treatment to tuberculosis (TB) development in high-risk patients, highlighting the challenge of managing NSCLC without compromising the immune system. Consequently, screening via IGRA and hepatitis B serology is recommended before starting ICIs to prevent the reactivation of latent TB or hepatitis [43].

Some guidelines, including those from the United States Preventive Services Task Force, recommend latent TB screening for patients at higher risk of developing active TB, which may include cancer patients prior to initiating immunotherapy [51].

Drug interactions between anti-TB and anticancer agents

Significant pharmacologic interactions during antituberculous therapy chiefly involve rifampicin, isoniazid, and fluoroquinolones. Rifampicin induces, whereas isoniazid inhibits cytochrome P450—mediated metabolism. Rifampicin interacts broadly with anticoagulants, contraceptives, and psychotropics; isoniazid affects anticonvulsants, theophylline, and paracetamol. Fluoroquinolones may alter absorption or enzyme activity. Elderly and immunocompromised patients warrant heightened surveillance [52].

LC and TB often coexist. Rifamycins are central to TB treatment, but their effects on hepatic metabolism of anticancer drugs can pose significant concerns [53]. It poses significant challenges due to drug–drug interactions. Its clinical impact includes autoinduction, which can reduce efficacy or cause treatment failure. Rifampicin can alter the metabolism or transport of coadministered drugs processed by cytochrome P450 enzymes or P-glycoprotein in the liver and gastrointestinal tract [54]. Since many anticancer drugs are CYP3A4 substrates, assessing interactions with CYP3A4 modulators like ketoconazole or rifampicin is standard in early development. Rifampicin drug–drug interactions are complex, involving CYP3A4 induction as the main driver for oral drugs, with contributions from P-glycoprotein, uridine 5′-diphospho-glucuronosyltransferase induction, and transporter inhibition. Paradoxically, CYP3A4 induction reduced exposure for intravenous drugs such as romidepsin and, to a lesser extent, cabazitaxel [55].

## 6. Conclusions

The interplay between tuberculosis and cancer is complex and bidirectional, with both conditions capable of mutually increasing the likelihood of co-occurrence. TB, as a chronic infectious disease, frequently induces extensive pulmonary tissue remodeling; the resulting inflammatory microenvironment may facilitate malignant transformation. Conversely, lung cancer and its treatments—particularly chemotherapy—can lead to myelosuppression and systemic immunosuppression, thereby heightening susceptibility to infections, including TB.

Although both pose major global health challenges, the coexistence of tuberculosis and lung cancer further increases diagnostic and therapeutic complexity. Multiple mechanisms may contribute to the development of active TB in patients with lung cancer, including reactivation of latent lesions due to disease- or therapy-related immunosuppression, as well as an increased risk of acquiring a new (“de novo”) infection.

Understanding these bidirectional interactions is essential for improving prevention, early detection, and integrated management strategies for patients affected by both conditions. Future research and clinical practice could be strengthened by routine latent TB infection screening in LC patients, particularly in endemic regions, prospective studies assessing TB reactivation risk during immune checkpoint inhibitor therapy, and the development of integrated management protocols for patients with concurrent TB and lung cancer.

## Figures and Tables

**Table 3:** Imaging Features of Pulmonary Tuberculosis, Lung Cancer, and Their Overlap.

Feature	Tuberculosis (TB)	Lung Cancer (LC)	Notes/Overlap
CT Appearance	Heterogeneous: cavitation, fibrosis, nodular infiltrates, often combined patterns [37]	Pulmonary nodules: solid or subsolid; features include spiculation, lobulation, vascular convergence, pleural retraction [38]	Both can present as nodules; TB can mimic malignancy
Nodule Density	Variable; may have calcification in chronic TB [37]	Solid or subsolid; solid nodules have higher malignant potential [38]	Overlap possible in granulomatous TB
Borders/Margins	Often irregular, ill-defined; may have fibrotic strands [37]	Spiculated or lobulated margins suggest malignancy [38]	Irregular TB lesions can resemble spiculated nodules
Lobe Involvement	Upper lobe predominance common; may involve multiple lobes [37]	Variable; can be peripheral or central; usually localized [38]	Both may be single or multiple lesions
Enhancement/Contrast Pattern	Variable enhancement; subjective assessment [39]	Often heterogeneous enhancement in malignant nodules [39]	Imaging alone may not differentiate reliably
Mediastinal Lymphadenopathy	Common in TB; may have central necrosis [40]	May occur in LC; often associated with nodal metastases [40]	PET uptake can be high in both
Functional Impact	Pulmonary function may range from normal to severely reduced depending on fibrosis or cavitation [37]	Usually minimal unless large or obstructive tumor [38]	TB causes more heterogeneous functional impairment
PET/FDG Uptake	High metabolic activity; tuberculoma and active lesions show uptake [38,40]	High metabolic activity; FDG uptake correlates with malignancy [40,41]	Both can be false positives; PET cannot reliably distinguish TB from LC
Diagnostic Consideration	Mimics malignancy; often requires microbiological confirmation [40]	Requires biopsy and histopathology for definitive diagnosis [36]	Misdiagnosis common; imaging alone insufficient
Key Challenge	Variable presentation; “diagnostic chameleon” [40]	Small lesions may lack classic malignant features [38]	Definitive differentiation often requires tissue confirmation

## Abbreviations

The following abbreviations are used in this manuscript:

TB	Tuberculosis
LC	Lung Cancer
NCDs	Non-Communicable Diseases
TBL	Tracheal, Bronchus, and Lung
DALYs	Disability-Adjusted Life Years
COPD	Chronic Obstructive Pulmonary Disease
PAF	Population Attributable Fraction
IRRs	Incidence Rate Ratios
CPI	Chronic Pulmonary Infection
ICIs	Immune Checkpoint Inhibitors
Mtb	Mycobacterium tuberculosis
TME	Tumor Microenvironment
TNF-α	Necrosis Factor-Alpha
IFN-γ	Type II Interferon
BCG	Bacille Calmette-Guérin
circRNAs	Circular RNAs
miRNAs	MicroRNAs
LTBI	Latent Tuberculosis Infection
TST	Tuberculin Skin Test
PTB	Pulmonary Tuberculosis
TAMs	Tumor-Associated Macrophages
